# Enhancing fitness, enjoyment, and physical self-efficacy in primary school children: a DEDIPAC naturalistic study

**DOI:** 10.7717/peerj.6436

**Published:** 2019-02-20

**Authors:** Francesca Vitali, Claudio Robazza, Laura Bortoli, Luciano Bertinato, Federico Schena, Massimo Lanza

**Affiliations:** 1 Department of Neurosciences, Biomedicine, and Movement, University of Verona, Verona, Italy; 2 Department of Medicine and Aging Sciences, “G. d’Annunzio” University of Chieti-Pescara, Chieti, Italy

**Keywords:** Physical activity, Health, Youngsters, Wellness

## Abstract

**Background:**

Physical activity (PA) (e.g., sport, physical education) promotes the psychophysical development of children, enhances health and wellbeing, offers opportunities for enjoyable experiences, and increases self-efficacy.

**Methods:**

In the DEDIPAC framework, we conducted a naturalistic, cross-sectional study to evaluate the effects of a school-based, long-term intervention on fitness (i.e., cardiovascular endurance, muscular strength, flexibility, speed, and finger dexterity), body mass index (BMI), PA levels, sedentary levels, enjoyment, and physical self-efficacy in primary school children. A group of Italian children (41 boys and 39 girls, aged 10–11 years) involved in the project—named “Più Sport @ Scuola” (PS@S)—was compared with a group of children (41 boys and 39 girls) of the same age not involved in the project.

**Results:**

After a four-year long attendance to the PS@S project, participants reported higher scores of cardiovascular endurance, muscular strength, enjoyment, and physical self-efficacy compared to children not involved in the project. Correlation analysis results showed that muscular strength scores correlated positively with BMI, PA levels, and enjoyment. Flexibility of the upper body was positively related to physical self-efficacy, and negatively related to speed and BMI.

**Conclusions:**

Findings suggest that the PS@S project enhanced fitness level, enjoyment, and physical self-efficacy of children.

## Introduction

The physical and psychological health benefits of regular physical activity (PA) in youth are well-documented (Janssen & LeBlanc, 2010; Poitras et al., 2016). Physically active youngsters were shown to have higher levels of fitness (e.g., stronger muscles, lower body fatness, stronger bones) (Carson et al., 2016; Cliff et al., 2016), enhanced psychosocial well-being (Hinkley et al., 2014), reduced symptoms of anxiety and depression (Doré et al., 2016), and a better chance to have a healthy adulthood (Janssen, 2007) compared to their inactive peers. Findings of extensive research have provided specific health-related recommendations for children and adolescents different from those for adults. For example, 5- to 17-year-olds are recommended to undertake moderate or vigorous PA for at least 60 min per day (US Department of Health and Services, 2008).

PA and sport are both included in leisure-time and school physical education. Sport, as an organized, competitive activity determined by rules and performed individually or in teams, plays an important role in the psychophysical development of children (Côté & Fraser-Thomas, 2007). Sport participation improves skills development, cardiorespiratory and cardiovascular functions, muscular strength, muscular endurance, speed, and flexibility (Boreham & Riddoch, 2001; Kristensen et al., 2010). Sporting activities also provide youth participants with enjoyable experiences and opportunities to increase self-efficacy and subjective well-being. A recent systematic review on children and adolescents (Eime et al., 2013) provided support to the psychological and social benefits of participation in sport, with the most commonly being improved self-esteem and social interactions, and reduced depressive symptoms. Children spend a considerable amount of time at school, which represents a powerful socializing agent having an important impact on establishing PA habits. Relatively few studies have investigated the specific role of school on PA promotion (Maes & Lievens, 2003; Zhu, 1997; Kristensen et al., 2013). Nevertheless, examining the impact of school in promoting healthy behaviors can set the scene for effective interventions aimed at fostering PA and contrasting sedentary behaviors.

The so-called Determinants of Diet and Physical Activity (DEDIPAC) was a multidisciplinary consortium of scientists established in 2013, involving several research centers in twelve countries across Europe. The DEDIPAC final report was delivered at the end of 2016. The main aim of the scientists of the DEDIPAC consortium was to understand the principal determinants of dietary behaviors, PA, and sedentary behaviors across the individual life course, and to translate this knowledge into a more effective promotion of healthy eating habits and an active life style for European citizens (Brug et al., 2017). Moreover, DEDIPAC contributed to (a) further PA measurement and monitoring; (b) provide overviews regarding the individual, social, and environmental determinants of dietary, PA, and sedentary behavior; and (c) create an online platform (http://www.dedipac.eu/toolbox) to include dataset, projects, and papers for researchers, practitioners, and policy makers for research and practical interventions. The platform is divided into four sections. The development section is aimed at guiding users through the process of developing a policy or multi-component intervention. The evaluation section contains guidelines and specific instruments geared toward evaluating policies and multi-component interventions. The implementation section provides information on the implementation and evaluation process. Finally, the natural experiments section offers practical examples of policies and multi-component interventions (Brug et al., 2017).

In the DEDIPAC framework, we conducted a naturalistic, cross-sectional study to examine the effects of the school-based, long-term intervention aimed at promoting PA for children. The project, named “Più Sport @ Scuola” (PS@S; “More Sport @ School”), was implemented in 2004—the European year of education through sport, an initiative launched by the European Union to promote a better use of sports as an educational and social inclusive tool—by the Veneto region (Italy) in collaboration with schools, local administrations, and sport clubs. The project lasted eleven academic years and ended in 2015. The central aim of the PS@S project was to increase the time dedicated to PA and sport at school. Primary school children were involved in a mean of 40 h of PA and sport activities across an academic year in addition to the customary physical education classes (i.e., 2 h a week). PA and sport activities were conducted by experts in collaboration with primary school teachers.

Several reviews have reported associations between PA and health and well-being outcomes among children (Poitras et al., 2016; Carson et al., 2016; Cliff et al., 2016). To date, relatively scarce attention has been directed towards the effects of school-based, long-term interventions aimed at promoting PA for children. A recent review examined the effects of PA across the activity spectrum (i.e., all waking behaviors, including sedentary behavior and low and high intensity activity patterns) and cardio-metabolic risk factors in children and adolescents (Verswijveren et al., 2018). Of the 15,947 articles identified, 29 were included in the review—24 studies were observational and 5 were experimental. None of these studies assessed the long-term effects of school-based interventions aimed to promote PA among children. Thus, the aim of the current study was to examine the effects of the school-based, long-term intervention PS@S project on fitness (i.e., cardiovascular endurance, muscular strength, flexibility, speed, and finger dexterity), body mass index (BMI), PA levels, sedentary levels, enjoyment, and physical self-efficacy of primary school children. The PS@S project was expected to enhance fitness and promote enjoyment and physical self-efficacy in participants.

## Materials & Methods

### Participants

The total sample consisted of 160 children aged 10–11 years (*M*_age_ = 10.42 years, *SD* = .23 years) attending primary school in North Italy. A subsample (41 boys and 39 girls) was involved in the PS@S project—a naturalistic, cross-sectional study—across four years. The other subsample of children (41 boys and 39 girls), involved as a control group, did not participate in the project. Students were drawn from intact classes of eight schools given that it was impossible, in a real-world school environment, to assign participants randomly to groups.

The study received approval from the Research Ethics Committee of the University of Verona (DEDIPAC WP3.2). Agreement to conduct the study was obtained from parents and headmasters of the schools after having explained them the general purpose of the study. Children provided written assent and their parents signed an informed consent. There were no parents or pupils who chose not to participate.

### Procedure

The children who participated in the PS@S project practiced a mean of 40 h of PA and sport across each academic year in addition to the customary physical education classes (i.e., 2 h a week). PA and sport activities were conducted by expert teachers in collaboration with primary school teachers. The children were involved in the PS@S project when 6- or 7-years old, and then were assessed after four years of participation when 10–11 years old. The control group took part to the customary physical education classes (i.e., 2 h a week for four years) conducted by the primary school teachers.

Assessment was conducted at school during physical education lessons. Two trained investigators administered the fitness tests individually, in presence of the teacher, during two sessions lasting approximately 40 min each. A multi-section questionnaire was also administered in groups of up to five children in quiet locations. Prior to questionnaire administration, participants were explained the general purpose of the study. They were also presented with instructions indicating that there were no right or wrong answers, and emphasis was placed on the confidentiality of responses.

### Measures

#### Fitness

Several fitness tests adapted from the Eurofit Physical Fitness Test Battery (Eurofit , 1988) were administered to participants. Cardiovascular endurance was assessed using the multi-stage 20 m shuttle run test, while muscular strength was measured by the standing broad jump. The sit-and-reach test and the back-scratch test were used respectively to evaluate flexibility of the lower back and hamstring, and of the upper body. Speed was measured through the Eurofit 10 ×  5 m shuttle run test. In addition, finger dexterity was measured with the nine-hole peg test.

#### Anthropometry

Weight in kilograms and height in meters were used to calculate the BMI. Since BMI is age- and sex-specific, we calculated it using the cut offs defined for children and adolescents (Cole et al., 2007).

#### Physical activity and sedentary levels

The Children’s Leisure Activities Study Survey (CLASS) questionnaire was used to assess the type, frequency, and duration of PA and sedentary behaviors of children (Telford et al., 2004). The reliability of the frequency and duration of children’s PA was previously examined using an intraclass correlation coefficient (ICC) and 95% confidence intervals, leading to reliable estimates of PA among 5- to 6-year-olds and 10- to12-year-olds (Telford et al., 2004). The reliability estimates of the CLASS questionnaires are consistent with other methodological studies showing retest correlations ranging from .60 to .79 for 7-day recalls with older children (Sallis & Saelens, 2000).

#### Enjoyment

The Italian version of the Physical Activity Enjoyment Scale (PACES) was used to evaluate individual enjoyment associated with PA and sports (Carraro, Young & Robazza, 2008). The scale includes sixteen statements scored on a 5-point Likert scale ranging from 1 = “*disagree a lot*” to 5 = “*agree a lot*”. The stem for each item is “When I am active …”. Nine items are positive (e.g., “I enjoy it”, “I find it pleasurable”) and seven items are negative (e.g., “I feel bored”, “I dislike it”). A total enjoyment score was obtained by reversing negative item scores and summing them to positive item scores. The two-factor solution was confirmed in an Italian sample across gender and age (from 11 to 19 years) using confirmatory factor analysis (Carraro, Young & Robazza, 2008). Cronbach *α* values ranged from .78 to .89.

#### Physical self-efficacy

Children’s perception of physical self-efficacy was assessed using the Perceived Physical Ability Scale for Children (Colella et al., 2008). This six-item scale evaluates the children’s perception of personal strength, speed, and coordinative abilities. Responses were indicated on a 4-point scale ranging from 1 = “*I run very slowly*” to 4 = “*I run very fast*”. Children were required to choose one of the four sentences best representing their personal feelings when playing, performing PA, or executing sporting activities. Items 1, 3, and 5 are scored from 1 to 4, whereas scores of items 2, 4, and 6 are reversed. Therefore, the total test score was calculated by reversing items 2, 4, and 6 and summing them to the remaining three items. Confirmatory factor analysis in an Italian sample supported the one-factor solution across gender and age (from 8 to 10 years). The Cronbach *α* value of the whole sample was .72 (Colella et al., 2008).

### Data analysis

Statistical analyses were performed using SPSS v.25. Data were screened for missing data, violations of assumptions of normality, linearity, multicollinearity, and homogeneity of variance–covariance matrices through frequency, scatter plots, and Box’s M test (Tabachnick & Fidell, 2013). Descriptive statistics and Pearson correlation coefficients were derived.

To examine possible differences between the two groups of children involved and not involved in the PS@S project on fitness (i.e., cardiovascular endurance, muscular strength, flexibility, speed, and finger dexterity), BMI, PA levels, sedentary levels, enjoyment, and physical self-efficacy, a 2 × 2 × 2 (group × gender × age) multivariate analysis of variance (MANOVA) was performed on the scores of all dependent variables. For all analyses, statistical significance was set at *p* < .05. Univariate follow-up was then conducted to determine the locus of significant differences.

## Results

Descriptive statistics are reported in Table 1. Significant MANOVA results were found by group, Wilks’ *λ* = .027, *F* (11, 142) = 471.199, *p* < .001, ${\eta }_{\mathrm{p}}^{2}=.973$, power = 1.000. Scores of cardiovascular endurance, muscular strength, and PA levels of the group of children involved in the PS@S project were higher than those of the group of children not involved in the project, whereas BMI scores and sedentary levels were lower (see Table 2). Significant differences were not observed on scores of the remaining physical capacities (i.e., flexibility, speed, and finger dexterity). Enjoyment and physical self-efficacy scores of the group of children involved in the PS@S project were also higher than those of the group of children not involved in the project.

**Table 1:** Descriptive statistics of fitness (cardiovascular endurance, muscular strength, flexibility, speed, and finger dexterity), BMI, PA and sedentary levels, enjoyment, and physical self-efficacy of primary school children in the two groups by gender and age.

	Group of children involved in the PS@S project								Group of children not involved in the PS@S project							
	Boys				Girls				Boys				Girls			
	10 yrs. (*n* = 22)		11 yrs. (*n* = 19)		10 yrs. (*n* = 18)		11 yrs. (*n* = 21)		10 yrs. (*n* = 20)		11 yrs. (*n* = 21		10 yrs. (*n* = 20)		11 yrs. (*n* = 19)	
	*M*	*SD*	*M*	*SD*	*M*	*SD*	*M*	*SD*	*M*	*SD*	*M*	*SD*	*M*	*SD*	*M*	*SD*
Endurance	33.17	1.29	34.13	1.06	32.87	0.82	33.02	1.02	29.90	0.32	30.74	0.65	28.40	0.59	30.07	0.62
Muscular strength	139.91	11.81	142.11	7.47	132.00	7.15	134.10	7.35	130.65	5.31	130.33	7.77	129.50	5.67	130.74	8.27
Flex. lower back	−3.91	5.66	−2.32	6.61	−0.94	2.73	−1.67	4.74	−1.75	3.14	−4.33	3.31	1.35	4.18	−3.32	2.94
Flex. upper body	4.41	5.40	0.32	4.42	6.00	4.61	3.24	2.95	3.85	4.84	2.86	3.68	4.40	3.20	2.58	4.56
Speed	20.78	1.48	23.28	2.14	21.01	2.06	24.60	2.69	23.82	6.59	21.93	1.25	23.44	1.63	22.20	2.01
Finger dexterity	20.08	3.10	20.31	2.96	19.79	2.17	20.00	2.74	19.85	4.11	20.23	2.23	19.61	2.91	19.78	2.68
BMI	18.35	0.39	18.77	0.35	17.23	0.37	18.27	0.49	19.51	0.44	20.53	0.61	18.38	0.32	19.51	0.73
PA levels	995.94	31.60	999.54	36.30	881.01	42.70	860.18	56.93	620.78	29.04	623.18	39.00	543.70	51.69	567.40	61.44
Sedentary levels	1,295.05	47.25	1,267.13	96.75	1,187.01	93.97	1,172.72	125.90	1,231.41	62.57	1,258.98	102.33	1,352.43	93.08	1,336.35	130.18
Enjoyment	4.50	0.28	4.44	0.28	4.47	0.28	4.43	0.26	3.90	0.34	3.72	0.40	3.88	0.42	3.90	0.46
Physical self-efficacy	3.42	0.27	3.30	0.25	3.41	0.31	3.36	0.30	2.13	0.26	2.08	0.29	2.25	0.25	2.17	0.27

**Table 2:** Pearson correlation coefficients (Sample 1, Sample 2).

Variables	1	2	3	4	5	6	7	8	9	10
1. Endurance	–									
2. Muscular strength	.38^b^, .12	–								
3. Flex. lower back	.11, −.43^b^	−.01, .08	–							
4. Flex. upper body	−.03, −.15	−.01, −.05	−.05, .02	–						
5. Speed	−.15, −.15	−.12, −.09	−.03, .01	−.32^b^, −.21	–					
6. Finger dexterity	−.06, .06	−.02, −.06	−.06, −.252^a^	.15, −.17	.20, .54^b^	–				
7. BMI	−.25^a^, .65^b^	.29^b^, −.02	−.09, −.44^b^	−.25^a^, −.10	.25^a^, −.09	.06, .10	–			
8. PA levels	−.18, .37^a^	.30^b^, .20	−.23^a^, −.25^a^	−.18, .05	−.25^a^, −.07	.08, −.08	.40^b^, .26^a^	–		
9. Sedentary levels	.13, −.34^a^	.02, .06	.05, .07	−.12, .24^a^	−.09, −.06	−.03, −.13	.21, −.29^b^	.38^b^, −.08	–	
10. Enjoyment	−.04, −.01	.23^b^, −.20	.06, .09	.11, .01	−.09, −.09	.10, −.25^a^	−.02, −.12	−.03, −.12	.07, .07	–
11. Physical self-efficacy	−.05, −.26^a^	.07, −.12	−.05, .13	.30^b^, .22^a^	−.13, −.10	−.03, −.20	−.01, −.18	−.17, −.22	−.04, .26^a^	.13, .45^b^

**Notes.**

Sample 1, Group of children involved in the PS@S project; Sample 2, Group of children not involved in the PS@S project).

a Low correlation.

b Moderate correlation.

Significant MANOVA results by gender were also found, Wilks’ *λ* = .218, *F* (11, 142) = 46.271, *p* < .001, ${\eta }_{\mathrm{p}}^{2}=.782$, power = 1.000. Cardiovascular endurance, muscular strength, flexibility scores of lower back and hamstring, BMI scores, and PA levels were larger in boys than girls (see Table 1). Finally, significant MANOVA results were observed by age (10 vs 11 years), Wilks’ *λ* = .385, *F* (11, 142) = 20.590, *p* < .001, ${\eta }_{\mathrm{p}}^{2}=.615$, power = 1.000. Cardiovascular endurance, flexibility, and BMI scores of 11-year-olds were higher than those of the 10-year-olds.

Pearson correlations coefficients are reported in Table 2. Of note, considering the group of children involved in the PS@S project, cardiovascular endurance scores correlated positively with muscular strength scores and negatively with BMI, while muscular strength scores correlated positively with BMI, PA levels, and enjoyment. Finally, flexibility of the upper body scores correlated positively with physical self-efficacy, and correlated negatively with speed and BMI.

## Discussion

Findings of the current naturalistic, cross-sectional study suggest that the four-year intervention (i.e., the PS@S project) in primary school was effective in enhancing children’s level of fitness, in particular cardiovascular endurance and muscular strength. Children involved in the PS@S project also showed higher levels of enjoyment associated with PA and sport compared to children not involved in the project, as well as higher levels of physical self-efficacy. These results are remarkable given the relatively limited research in the school setting aimed to promote PA and sport participation among primary school children (Maes & Lievens, 2003; Zhu, 1997; Kristensen et al., 2013). Effective intervention strategies can promote PA and sport not only in the school years, but also encourage students to adopt PA as a pleasant healthy habit in leisure time and adulthood. Indeed, extant literature indicates that PA preferences and habits established in childhood tend to persist into later life stages (Kristensen et al., 2008).

An additional important finding is the significant difference observed in the BMI scores of children involved in the PS@S project which were lower compared to children who did not participate in the project. This result is of particular interest given the serious health consequences associated with overweight and obesity that are progressively increasing and spreading out in Italy (Istituto Superiore di Sanità, 2016; Sacchetti et al., 2012) and in several European countries (Brug et al., 2010; Brug et al., 2012). The school can be an ideal setting to promote PA and prevent children’s overweight prevalence.

Finally, the two groups of children differed in terms of PA levels. Children who took part in the PS@S project reported higher PA scores than their peers not involved in the project. This result highlights the role of the school in promoting PA and sport for youth. School is a powerful socializing agent in children’s lives and can have an important impact on establishing PA habits. Thus, there is a clear need of national intervention strategies to promote PA and sport among primary school children.

Taken together, the purposes and findings of the current study conducted within the DEDIPAC framework are consistent with the mission of the project itself, namely, to assess PA habits and changes in PA behavior in targeted populations for policy interventions, and to learn from successes and failures of interventions in order to enhance the effectiveness of future projects across Europe and other counties (Brug et al., 2017). According to Brug et al. (Brug et al., 2017), there is a clear need of a better cross-European harmonization of assessment and monitoring procedures, and to collect longitudinal data to gain more insight into behavioral determinants of heathy behaviors.

## Conclusions

Despite the appeal of our findings, we acknowledge some limitations in the current study. First, the relatively small number of participants involved can limit the generalization of the results. Schools were difficult to recruit, and this is a common issue of school-based research in European countries that emerged in recent years (Brug et al., 2010). The emphasis placed by the governments and public health agencies on youth health promotion should be accompanied by interventions at school that are in accordance with a sound theoretical framework. Nevertheless, some schools are reluctant to participate. Second, the current study was conducted in a naturalistic setting. An advantage of this type of research is that it allows the investigators to directly observe and measure the participants in a natural environment with a higher external validity compared to a laboratory context. However, this method has also some potential downsides that must be considered, first of all the low control on measured variables can make difficult to determine the specific causes of a behavior. Moreover, in a real-world environment it is not possible to control the many confounding factors that may influence the study variables.

In conclusion, as expected the PS@S project was effective in enhancing fitness, in particular cardiovascular endurance and muscular strength, as well as enjoyment and physical self-efficacy. These beneficial effects are predicted to have long-term positive consequences in the adoption of an active lifestyle in adulthood (Di Battista et al., 2018). Future longitudinal research should examine more in detail the long-term impact of PA interventions at school in promoting healthy behaviors among children.

##  Supplemental Information

10.7717/peerj.6436/supp-1 Supplemental Information 1 Dataset_Dedipac_study

## Additional Information and Declarations

### Data Availability

The following information was supplied regarding data availability: Raw data are available as a Supplemental File.
